# Editorial: Measurement Tools for Clinical Assessment, Characterization and Neurorehabilitation of Parkinson's Disease

**DOI:** 10.3389/fneur.2021.700581

**Published:** 2021-05-28

**Authors:** Carmen Rodriguez-Blazquez, Maria João Forjaz, Mayela Rodriguez-Violante

**Affiliations:** ^1^National Epidemiology Center, Carlos III Health Institute, Madrid, Spain; ^2^CIBERNED, Madrid, Spain; ^3^REDISSEC, Madrid, Spain; ^4^Movement Disorder Clinic, National Institute of Neurology and Neurosurgery, Mexico City, Mexico

**Keywords:** Parkinson's disease, assessment, neurorehabilitation, endpoint, digital

## Introduction

As editors of this Research Topic on Measurement Tools for Clinical Assessment, Characterization and Neurorehabilitation of Parkinson's Disease, we are delighted to introduce the final collection of papers featured in this Research Topic.

In recent years, a wide variety of measurement tools have been developed for assessing motor and non-motor manifestations of Parkinson's disease (PD). Rater-based interviews and patient self-assessments provide an approximation to subjective and non-observable aspects of PD. The goal of this Research Topic is to offer a review on the recent advances in subjective and objective measurement tools for clinical assessment, characterization and neurorehabilitation of PD.

The topic is divided into four broad categories. The first one covers methodological studies on the psychometric properties of rating scales and questionnaires for clinical assessment, characterization and neurorehabilitation of PD. The second category comprises studied on validation of digital endpoints for clinical assessment, characterization, and neurorehabilitation of PD. The third category included studies on new developments and application of subjective and objective tools. Lastly, the fourth category is related to studies about the responsiveness and interpretation of change of measurement tools.

The final collection is comprised of 20 high-quality papers including 11 original research manuscripts, four systematic reviews, one review, and four brief research reports.

## Studies on Methodological Issues

Concerning non-motor aspects of PD, Fleury et al. presented a new scale to assess embarrassment and shame of people with PD. The SPARK scale, validated in a sample of 102 PD patients, provides valid and reliable data that may be of great usefulness to assess the social impact of PD.

Cognitive impairment is a common disabilitating non-motor PD, and two Spanish teams presented results from their research that can guide diagnosis and treatment. Specifically, the study by Horta-Barba et al. shows that, in a sample of 114 PD patients and 41 healthy controls, encoding and retrieval deficits are an important characteristic of mild cognitive impairment in PD patients, and it is associated with damage in specific brain areas. According to the study by Simon-Gozalbo et al., cognitive impairment in PD is associated with a more advanced disease stage and impact in psychosocial and quality of life outcomes.

Smell deficits and anosmia is one of the most common non-motor symptoms in PD, and could precede the onset of the motor symptoms. Zhao et al. explored the discriminatory power of the olfactory tests for the early diagnosis of PD, using Sniffin' Sticks test. The use of the identification domain test alone for assessing olfactory deficits in PD has great implications for clinical practice and research.

Gan et al. have studied the prevalence and clinical features of freezing of gait (FOG) in a sample of 838 PD patients. This study highlights the need of regularly assess gait and balance in PD patients, optimize the pharmacological treatment and implement early gait rehabilitation training.

## Studies on Digital Endpoints

In 16 consecutive patients, Béreau et al. showed that the software model was of limited use and results favored the clinical testing to select stimulation parameters. A review of technology-enabled care in PD presents it as a field with a lot of potential to provide comprehensive care and reduce health inequalities, although it also faces some challenges, such as the lack of standards of validation (Luis-Martínez et al.). An example of telerehabilitation intervention is provided by Isernia et al. with promising results.

A study by Wang et al. indicates that PD developed from essential tremor has specific characteristics, such as the presence of hyposmia and electrophysiological biomarkers, including postural tremor frequencies and amplitudes. In the treatment of PD, subthalamic nucleus deep brain stimulation requires determination of thresholds that can be clinically or software-guided.

## Studies on New Developments

The issue contains studies on new developments and application of subjective and objective tools. Temporiti et al. shares a systematic review on the Action Observation Therapy (AOT) based on the mirror neuron system. Overall, seven studies were included revealing that AOT is effective in improving walking ability, freezing of gait and bradykinesia. Nevertheless, study heterogeneity should be considered.

Sanderson et al. address the use of goal-directed tablet-based task to characterize the motor features of PD. The authors developed a touchscreen tablet-based motor task with continuously-moving target designed to capture goal-directed movement. Their tablet-based task and analysis protocol correlated strongly with expert clinical assessments using the MDS-UPDRS-III.

Knudson et al. provide a study aimed at comparing objective and subjective measures using the tool Parkinson's KinetiGraph (PKG). They included 34 patients who wore the PKG for 6 days during the normal daily activities and then used the data collected to build a regression model to predict the MDS-UPDRS II score achieving a significant correlation.

This section includes three papers on potential biomarkers. Ohmichi et al. assessed caffeine concentrations in serum and plasma as a potential blood-based biomarker for PD. They included persons with PD, persons with other parkinsonism, and healthy controls. They found decreased blood concentrations of caffeine in PD compared to controls with a similar trend in the multiple system atrophy group, which warrants further study.

Maycas-Cepeda et al. assessed the role of amimia as a potential marker of other motor and non-motor features of the disease. They included 75 persons with PD and correlated their UPDRS sub scores. They report that amimia correlates with axial symptoms and cognitive situations in PD.

Ramezani et al. analyzed the association between the p.Val66Met, a polymorphism in the BDNF gene, and mild behavioral impairment. Met carriers had a 2-fold likelihood of having mild behavioral impairment than Val carriers suggesting this allele is associated with a higher neuropsychiatric burden in PD.

## Studies on Responsiveness and Interpretation of Change

Finally, a group of papers focused on studies about the usefulness, responsiveness, and interpretation of change, due to either time or treatment, of measurement tools used in clinical assessment and characterization and neurorehabilitation of PD.

Wang et al. analyzed how deep brain stimulation (DBS) modulates the intraoperative neuromuscular pattern of resting tremor in 39 PD patients. They identified three intraoperative biomarkers that allow to quantify and predict the efficacy of DBS in PD patients with resting tremor in a quick and efficient way.

Balance dysfunction in PD is usually not respondent to pharmacological or surgical treatment. Hasegawa et al. have assessed the efficacy of a specific intervention for balance deficits, using objective (wearable sensors) and clinical and subjective measures. The authors recommend the use of the objective measures for assessing the efficacy of exercise in improving the balance deficits.

The study of the changes in echogenicity in brainstem raphe (BR) detected by transcranial parenchymal sonography (TCS) and their association with motor and non-motor symptoms in PD patients is the aim of the work by Bei et al. Their findings support the hypothesis of a pathogenetic link between depression and, combined with substantia nigra hyperechogenicity, might be useful to detect individuals at risk for developing PD.

The ability of neurovestibular laboratory tests to predict future falls in patients with PD or atypical parkinsonism (AP) was explored by Venhovens et al. Accurate determination of the risk factor of falls could reduce their incidence and the associated disease burden. Cervical and ocular vestibular evoked myogenic potentials (VEMP) combined with clinical tests for postural imbalance predicted future fall incidents in both PD and AP groups with a sensitivity of 100%.

Bouça-Machado et al. paper aimed at identifying which kinematic and clinical outcomes changes predict functional mobility (FM) changes in PD patients as a result of a specialized multidisciplinary program. These findings support the use of kinematic outcome measures to evaluate the efficacy of multidisciplinary interventions for FM in PD patients.

## Conclusions

In this collection of articles, authors have presented important developments and applications in objective and subjective measurement tools for PD. The development of new treatments for PD requires reliable and sensitive measurements of patients' health status and abilities of daily living. Validated rating scales and questionnaires are being adapted to digital devices that allow a constant monitoring of the clinical condition. Mobile or residential technology is implemented for remote assessment of health-related parameters or for rehabilitation purposes, and digital endpoints are being used in clinical trials. This field of research is constantly evolving and we will see further advances in the future.

## Author Contributions

All authors listed have made a substantial, direct and intellectual contribution to the work, and approved it for publication.

## Conflict of Interest

The authors declare that the research was conducted in the absence of any commercial or financial relationships that could be construed as a potential conflict of interest.

